# Inhibitory Effect of a Novel Ophthalmic Solution on *Acanthamoeba castellanii* Adhesion and Biofilm Formation on Human Corneal Epithelium

**DOI:** 10.3390/life15111685

**Published:** 2025-10-30

**Authors:** Francesco D’Oria, Giovanni Petruzzella, Daniel Narvaez, Marta Guerrero, Fedele Passidomo, Enzo D’Ambrosio, Francesco Pignatelli, Giuseppe Addabbo, Giovanni Alessio

**Affiliations:** 1 Section of Ophthalmology, Department of Translational Biomedicine and Neuroscience, University of Bari, 70121 Bari, Italy; 2 Retinset S.L., Amposta 20, 08174 Sant Cugat del Vallès, Spain; 3 Department of Pharmacy and Pharmaceutical Technology and Physical Chemistry, University of Barcelona, 08028 Barcelona, Spain; 4 Centro Oftalmico d’Ambrosio, 74121 Taranto, Italy; 5 Eye Clinic, “SS. Annunziata” Hospital, ASL Taranto, 74100 Taranto, Italy

**Keywords:** *Acanthamoeba* keratitis, corneal epithelium, CORNEIAL MED, amoebicidal activity, biofilm, preventive therapy

## Abstract

**Background/Objectives:** *Acanthamoeba* keratitis (AK) is a rare but sight-threatening corneal infection, often associated with contact lens wear and resistant to conventional therapies. Preventive strategies capable of reducing *Acanthamoeba* adhesion to corneal epithelium may represent an important tool for infection control. This study aimed to evaluate the amebicidal and preventive activity of CORNEIAL MED eye drops against *Acanthamoeba castellanii* adhesion and early adhesion layer on human corneal epithelium (HCE). **Methods:** Reconstructed HCE models were exposed to *A. castellanii* under four experimental conditions: negative control (HCE only), positive control (HCE + *A. castellanii*), co-incubation with CORNEIAL MED and *A. castellanii* (Study 1), and treatment with CORNEIAL MED after initial *A. castellanii* adhesion (Study 2). Adherent amoebae were quantified using EDTA detachment and Neubauer chamber counting. The early adhesion layer was characterized by scanning electron microscopy (SEM). Statistical analysis considered *p* < 0.05 as significant. **Results:** In Study 1, simultaneous application of CORNEIAL MED with *A. castellanii* reduced amoeba adhesion by 33.0 ± 11% compared with controls (*p* = 0.0529). In Study 2, when the product was applied 3 h after amoeba inoculation, adhesion was significantly reduced by 51.9 ± 6.5% (*p* < 0.05). SEM confirmed a decrease in amoebic colonization and biofilm density in treated samples. **Conclusions:** CORNEIAL MED demonstrated a measurable inhibitory effect on *A. castellanii* adhesion to HCE, particularly when applied after initial pathogen contact. These findings suggest a potential preventive role of CORNEIAL MED in reducing AK risk, although further in vivo studies are warranted.

## 1. Introduction

*Acanthamoeba* keratitis (AK) is a rare but vision-threatening corneal infection that disproportionately affects contact lens (CL) wearers and is tightly linked to lapses in lens hygiene and water exposure. Contemporary reviews emphasize that most AK cases arise in CL users, and that behaviors such as showering or swimming while wearing lenses confer substantial additional risk [1,2]. The early clinical picture is often nonspecific and overlaps with viral or bacterial keratitis, driving diagnostic delay and progression to deeper stromal disease with ring infiltrates, perineural keratitis, and—in severe presentations—scleritis and a need for keratoplasty [2].

In parallel, global CL wear continues to expand, with ~4.5 million users in the United Kingdom and >41 million in the United States [3,4]. This sustained exposure environment contributes to the worldwide burden of microbial keratitis (MK), estimated at 1.5–2.0 million cases annually [5]. The pathogen spectrum is broad, ranging from Gram-positive bacteria (e.g., *Staphylococcus aureus*) and Gram-negative bacteria (e.g., *Pseudomonas aeruginosa*) to fungi (e.g., *Candida albicans*, *Fusarium keratoplasticum*) and free-living amoebae (*Acanthamoeba* spp.)—with CL wear a prominent risk factor for several of these etiologies [3]. Among CL modalities, weekly/monthly soft lenses carry a particularly high risk due to overnight wear, suboptimal hand hygiene, case contamination, and variability in the effectiveness of multipurpose solutions (MPS) [6].

MPS are expected to meet rigorous performance standards for activity against bacteria and fungi (ISO 14729) and *Acanthamoeba* (ISO 19045-1); nonetheless, anti-amoebic efficacy remains heterogeneous across products and formulations [7,8,9,10]. To address this variability, newer systems combine biguanides with quaternary ammonium compounds (e.g., alexidine or polyaminopropyl biguanide, PAPB, paired with polyquaternium-1), and recent work has explored alternative guanidino-containing antiseptics such as polyhexamethylene guanide (PHMG) and guazatine for both CL disinfection and potential therapeutic roles [10,11].

Despite advances in diagnostics and care pathways, AK management remains challenging. First-line medical therapy typically relies on intensive topical biguanides—most commonly polyhexamethylene biguanide (PHMB) or chlorhexidine—either as monotherapy (e.g., 0.02%) or combined with diamidines (propamidine or hexamidine), often for months [2,12]. These regimens can be effective but are frequently limited by ocular surface toxicity, adherence burden, and prolonged time-to-cure. Contemporary randomized evidence from the ODAK trial supports the central role of biguanides, evaluating PHMB 0.08% against comparator regimens and reinforcing that biguanide-based therapy can achieve high medical cure rates in AK [12]. In parallel, preclinical and methodological studies continue to screen candidate amebicidal/cysticidal agents—including azoles and other chemical classes—to shorten therapy and improve tolerability [13]. However, therapeutic success is constrained by the organism’s ability to encyst, which confers striking drug tolerance and underlies relapses during tapering; corticosteroids, while sometimes necessary to control inflammation, must therefore be introduced judiciously [2].

From a pathogenic perspective, the earliest steps of infection involve trophozoite adhesion to corneal epithelium—mediated, in part, by a mannose-binding protein—followed by epithelial injury, inflammation, and encystment [14]. These mechanisms elevate epithelial adhesion and very-early colonization as rational yet underexploited targets for prevention and pre-emptive intervention. In vitro comparative microbiology indicates that PHMB and related guanidino polymers (PAPB, PHMG) exhibit potent activity against *Acanthamoeba* trophozoites and cysts and strong activity against *P. aeruginosa*, *S. aureus*, and *C. albicans*, whereas other candidates (e.g., guazatine) show more variable performance across organisms and conditions [10,11].

Formulation science may further enhance preventive potential at the ocular surface. Cross-linked hyaluronic acid (HA-CL) increases mucoadhesion and residence time while supporting epithelial health and re-epithelialization, thereby improving drug–surface contact at tolerable concentrations [15]. Building on this rationale, a PHMB 5 ppm, HA-CL–containing ophthalmic solution demonstrated broad antimicrobial activity in vitro (including fungistatic effects against *Candida albicans* and *Aspergillus fumigatus*) and reduced conjunctival bacterial load in vivo in peri-cataract settings, supporting the safety and antiseptic potential of this platform and its translational applicability from bench to clinical practice [16]. Formulation strategies (e.g., polymer cross-linking, viscosity modulation, surface interactions) can enhance ocular residence time and drug–surface contact, potentially improving early-phase pathogen control [17].

In this contest, the present study evaluates whether a PHMB 5 ppm, HA-CL–containing eye drop (CORNEIAL MED^®^) (Bionativa S.p.A., Florence, Italy) can reduce *Acanthamoeba castellanii* adhesion and early colonization on reconstructed human corneal epithelium (HCE). Using co-incubation (prophylaxis-mimicking) and post-adhesion (very-early intervention) paradigms, we quantify adherent trophozoites and assess surface architecture by scanning electron microscopy. We hypothesize that the formulation will measurably decrease epithelial adhesion and early biofilm-like organization, providing proof-of-concept for a tolerable preventive adjunct in high-risk scenarios such as soft CL wear.

## 2. Materials and Methods

**Figure 1:**
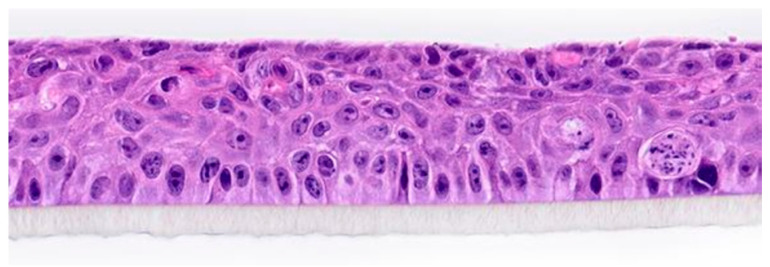
Histology of in vitro reconstructed human corneal epithelium (Episkin).

This study was conducted using a reconstructed HCE model (SkinEthic HCE/Corneal Epithelium, Episkin, Lyon, France) at the GAIKER Technology Center (Basque Research and Technology Alliance, Zamudio, Spain). Prior to experimentation, the samples were maintained in culture medium for 24 h at 37 °C under 5% CO_2_ to ensure epithelial stability (Figure 1).

The protozoan used was *Acanthamoeba castellanii* strain CCAP 1501/10, obtained from the Culture Collection of Algae and Protozoa (CCAP, SAMS Ltd., Oban, UK). Subcultures were prepared on non-nutrient agar (NN agar) plates inoculated with *Escherichia coli* and incubated aerobically for 7 days at 37 °C. A working suspension was then prepared in SMM+ medium, and the concentration was adjusted to 5 × 10^6^ cells/mL for use in the experiments. A total of 20 HCE samples were divided into four experimental groups, each consisting of five replicates.

- The negative control group (HCE) consisted of tissue inserts maintained under identical culture conditions without exposure to either amoebae or the test product. This group allowed verification of the baseline integrity of the epithelial model and excluded the possibility of spontaneous contamination or unspecific morphological alterations induced by the culture medium alone.
- The positive control group (HCE + AC) was designed to reproduce the natural course of infection. In this condition, HCE tissues were inoculated with *A. castellanii* trophozoites (10 µL suspension at 5 × 10^6^ cells/mL) and incubated for 24 h at 37 °C, 5% CO_2_. This group provided the reference standard for maximal amoebic adhesion in the absence of any treatment.
- The first study group (HCE + TP + AC, co-incubation paradigm) was intended to simulate a prophylactic scenario, in which CORNEIAL MED was present on the ocular surface at the time of amoebic challenge. A volume of 20 µL of CORNEIAL MED was applied to the apical surface of the epithelium, immediately followed by 10 µL of *A. castellanii* suspension. The tissues were then incubated for 24 h under standard conditions. This setup assessed the capacity of the product to interfere with the initial steps of trophozoite adhesion and colonization.
- The second study group (HCE + AC + TP, post-adhesion paradigm) was designed to mimic an early therapeutic intervention, corresponding to the clinical situation in which amoebae have already established initial contact with the corneal epithelium. In this protocol, HCE tissues were first inoculated with *A. castellanii* suspension and maintained for 3 h to allow adhesion. After this period, 20 µL of CORNEIAL MED was applied to the surface, and the tissues were incubated for an additional 24 h. This group enabled the evaluation of the inhibitory effect of the product once adhesion had already occurred.

At the end of each incubation period, the number of adherent amoebae was quantified. Cells were detached from the HCE surface using 2 mM EDTA in PBS at 4 °C for 15 min, then counted in a Neubauer chamber by optical microscopy. Results were expressed as the mean number of adherent cells ± standard deviation (SD), and also as the percentage reduction in adhesion compared with the positive control. For morphological characterization, samples were processed for SEM, using a Hitachi S-4800 FEG Scanning Electron Microscope (Hitachi Ltd., Tokyo, Japan). After fixation with 2% glutaraldehyde in 0.1 M phosphate buffer, samples were washed with the same phosphate buffer. Thereafter, samples were dehydrated through increasing concentrations of ethanol (30–50–70–90–100%) and dried with hexamethyldisilazane. Finally, samples were sputter-coated with a thin gold layer under an argon atmosphere. Images were acquired at magnifications of 300×, 1000×, and 5000× to evaluate surface colonization and biofilm architecture.

Data analysis was performed by comparing the treated groups with the positive control. Results are reported as mean ± SD. Statistical significance was assessed using Student’s *t*-test for pairwise analysis and ANOVA for multiple comparisons, with *p* < 0.05 considered significant.

## 3. Results

### 3.1. Preventive Effectiveness Study (Study 1)

When CORNEIAL MED was applied simultaneously with A. castellanii, the mean number of adherent trophozoites recovered from HCE surfaces was 11,062 ± 1819 cells/insert (95% CI 8169–13,956), compared with 14,250 ± 5489 cells/insert (95% CI 5516–22,984) in the positive control (HCE + AC). This corresponded to a 32.95 ± 11.02% mean reduction in adhesion relative to the positive control (reported *p* = 0.0524; Table 1). The trend indicates measurable inhibition in the prophylaxis-mimicking setting.

### 3.2. Preventive Effectiveness Study (Study 2)

**Figure 2:**
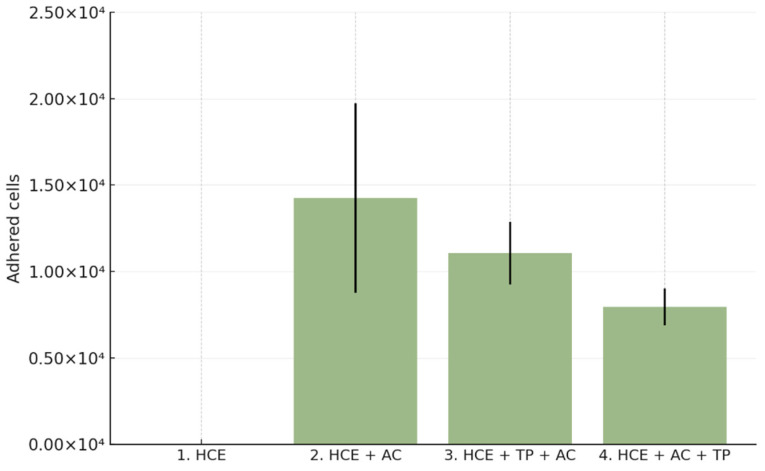
Quantitative analysis of *Acanthamoeba castellanii* adhesion to reconstructed human corneal epithelium (HCE). Bar chart shows the mean number of adherent trophozoites (±SD) across experimental groups: negative control (HCE), positive control (HCE + AC), co-incubation with CORNEIAL MED (HCE + TP + AC), and post-adhesion treatment (HCE + AC + TP). Both treatment paradigms reduced adhesion compared with the positive control, with a greater effect observed in the post-adhesion setting.

When CORNEIAL MED was applied 3 h after inoculation (post-adhesion paradigm), the mean number of adherent cells decreased to 7938 ± 1068 cells/insert (95% CI 6238–9637) versus 14,250 ± 5489 in the positive control, corresponding to a 51.89 ± 6.47% mean reduction in adhesion (reported *p* = 0.0073; Table 1). Quantitative SEM analysis further supported a marked decrease in surface colonization (Figure 2).

**Figure 3:**
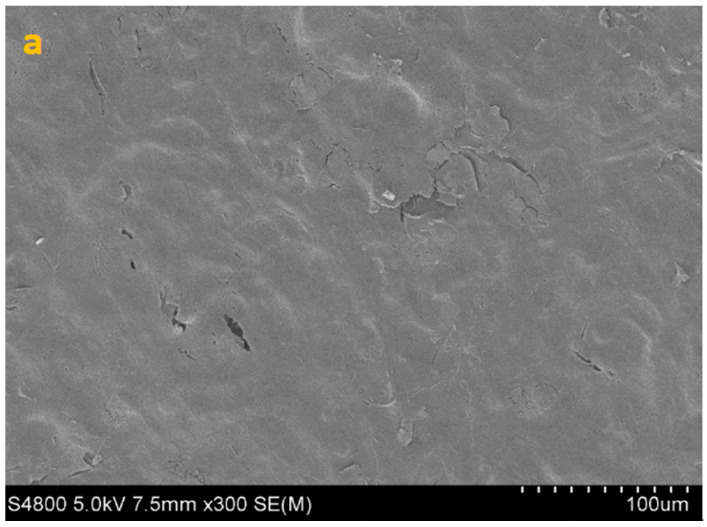

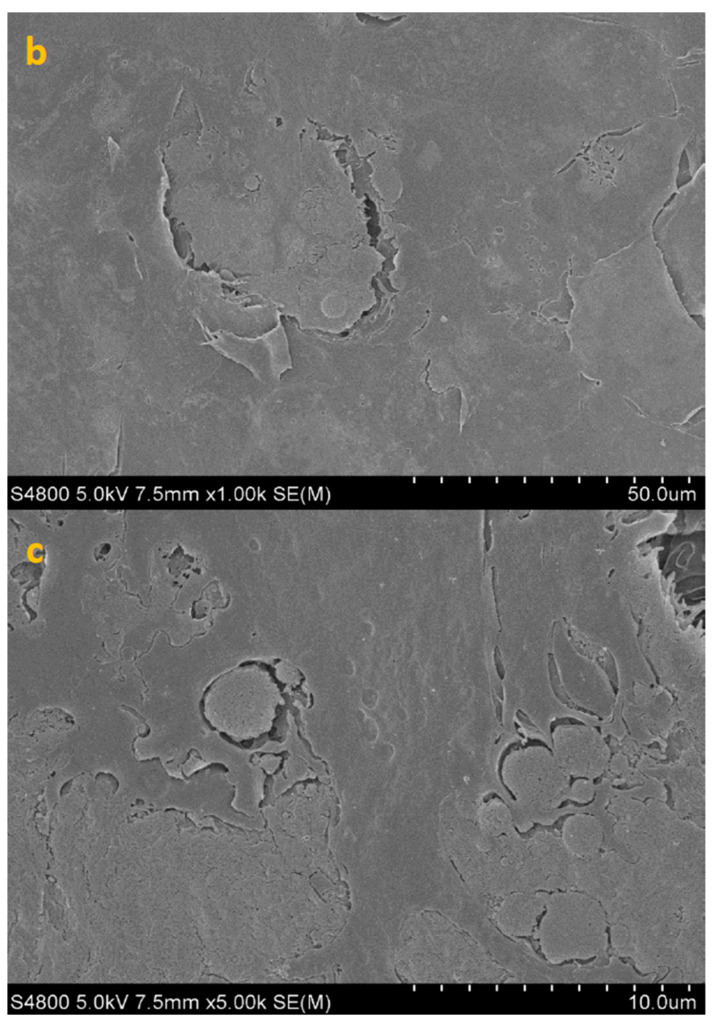
SEM image of HCE surface at 300× (**a**), 1000× (**b**) and 5000× (**c**) magnification.

**Figure 4:**
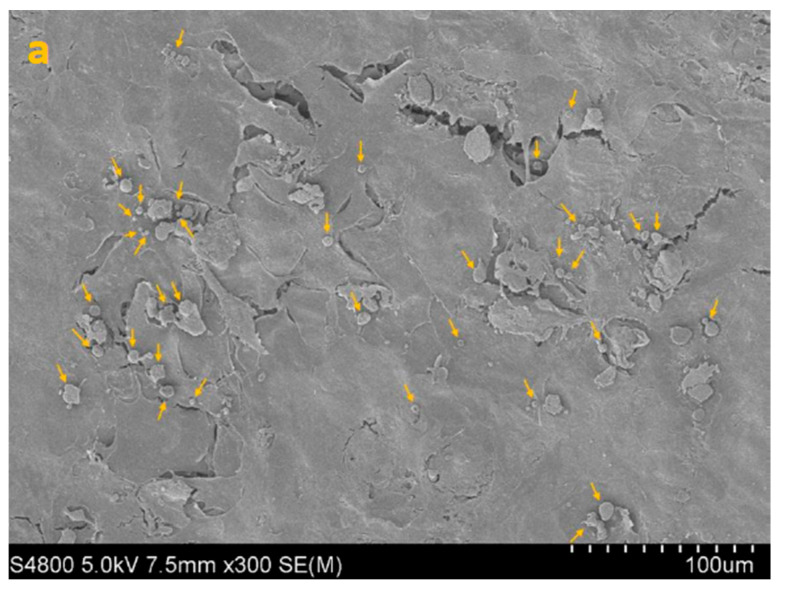

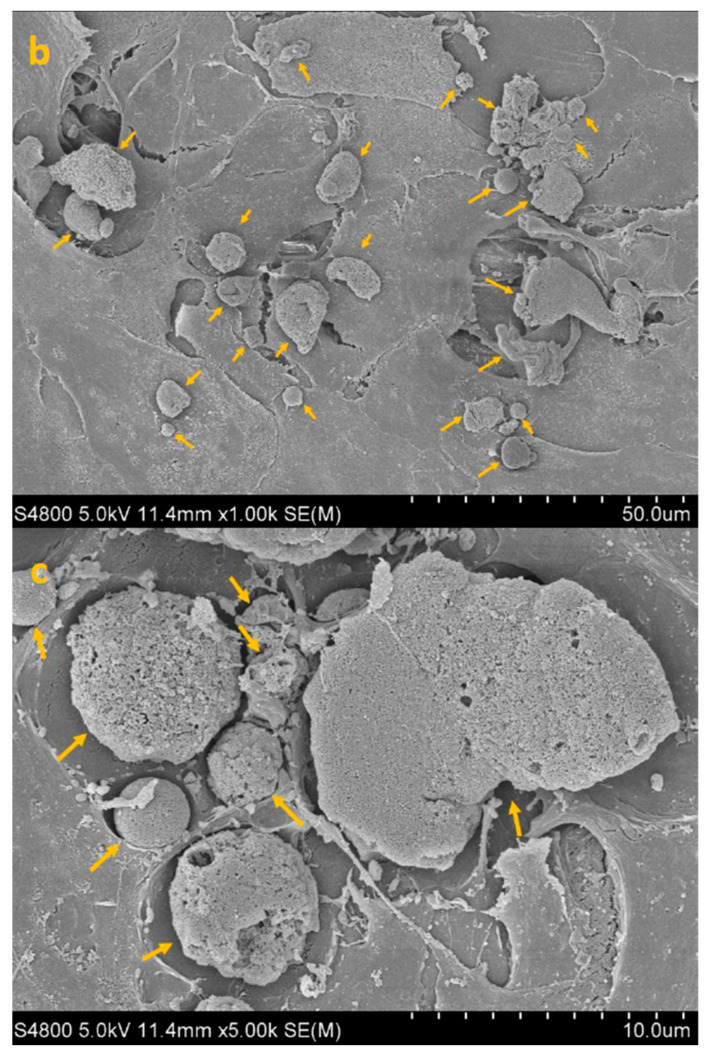
SEM image of HCE + AC at 300× (**a**), 1000× (**b**) and 5000× (**c**) magnification. Yellow arrows highlight densely adherent *Acanthamoeba* trophozoites forming the early adhesion layer.

**Figure 5:**
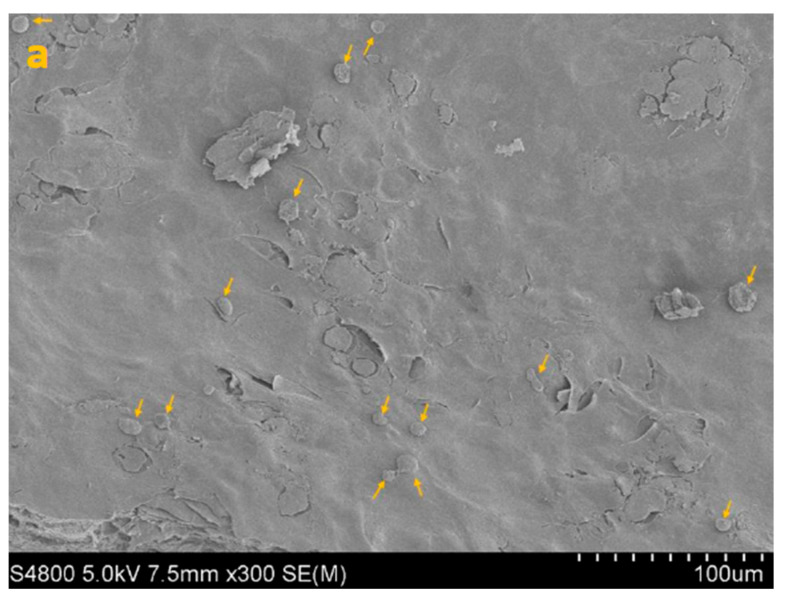

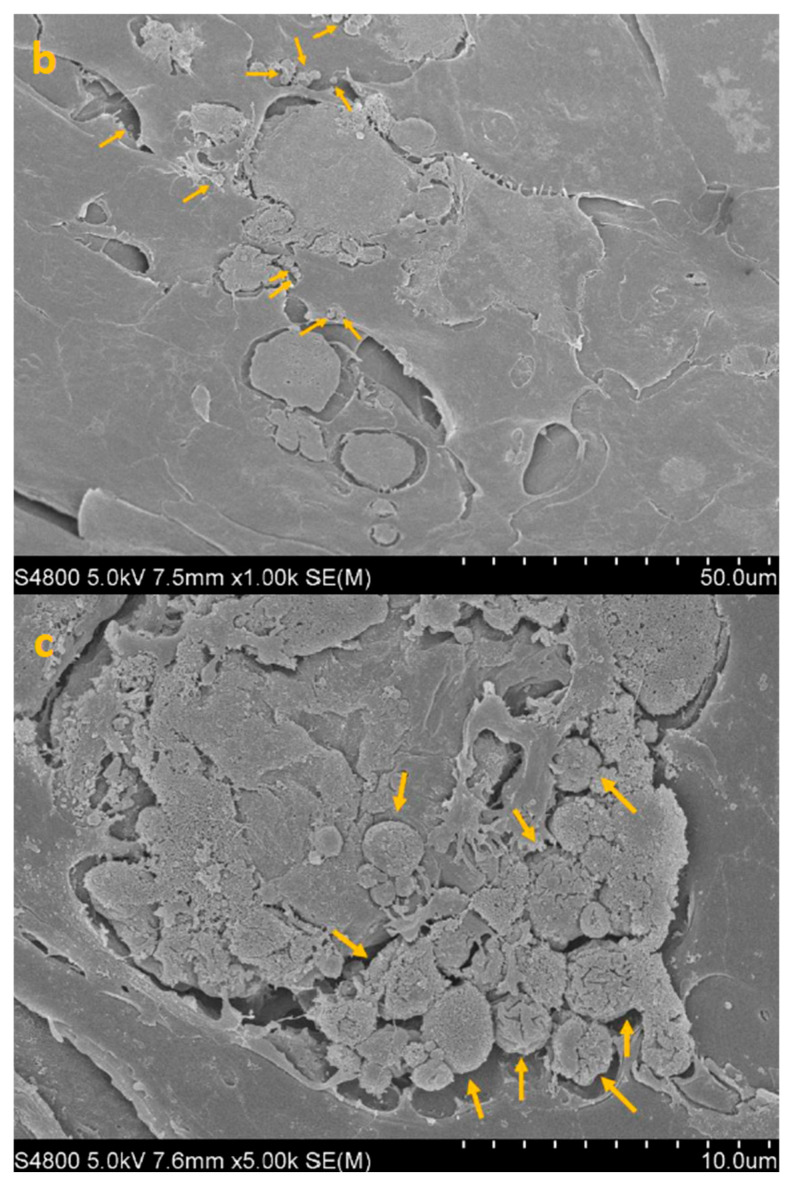
SEM image of HCE + TP + AC (co-incubation paradigm) at 300× (**a**), 1000× (**b**) and 5000× (**c**) magnification. Yellow arrows point to isolated trophozoites visible on the epithelial surface after co-incubation with CORNEIAL MED.

**Figure 6:**
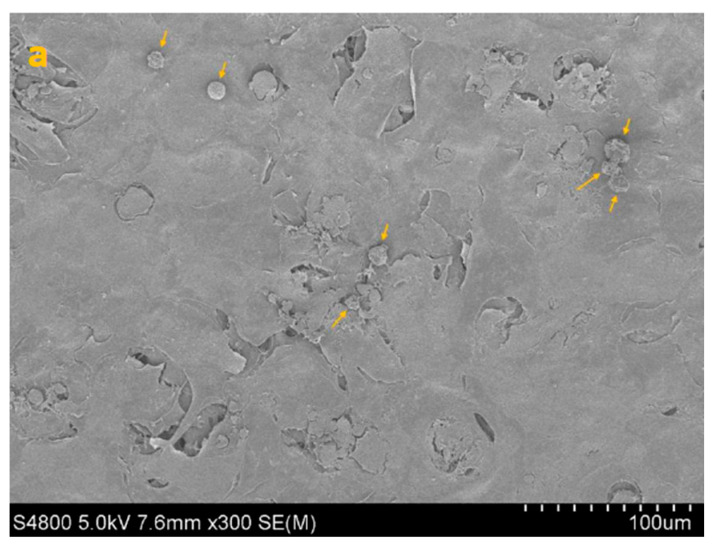

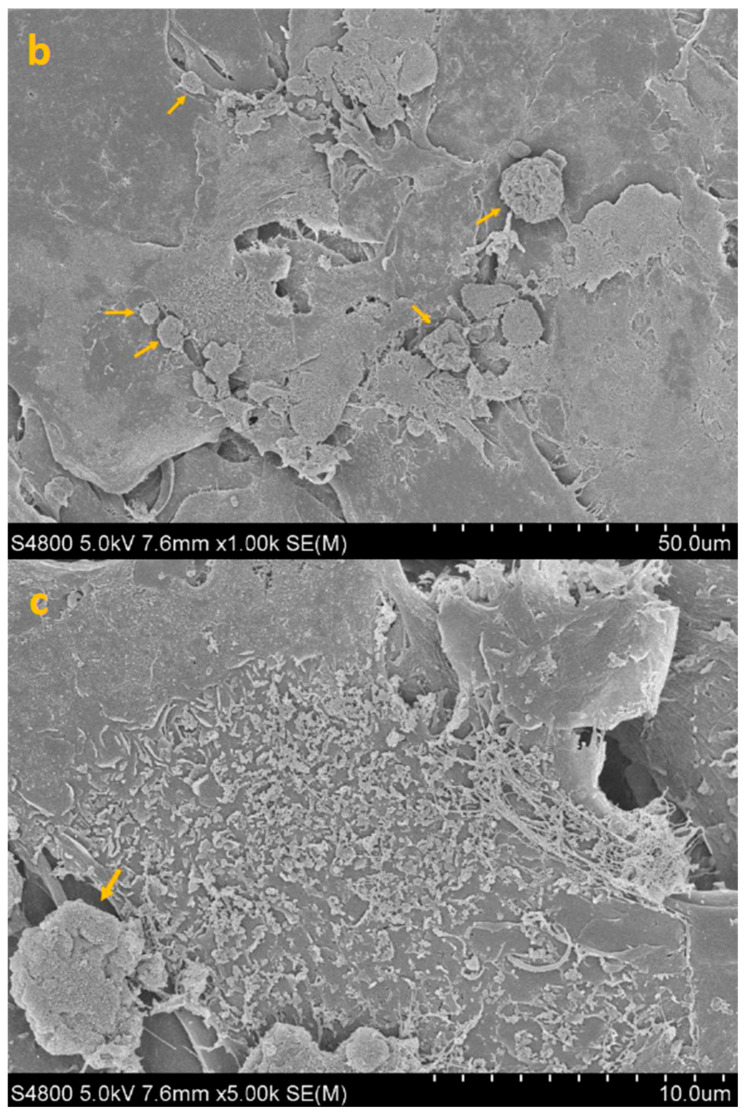
SEM image of HCE + AC + TP (post-adhesion paradigm, treatment at 3 h) at 300× (**a**), 1000× (**b**) and 5000× (**c**) magnification. Yellow arrows identify sparse trophozoites remaining following post-adhesion treatment with CORNEIAL MED.

SEM imaging corroborated the quantitative data, showing sparser colonization with isolated trophozoites and decreased surface coverage in treated samples (Figure 3, Figure 4, Figure 5 and Figure 6).

## 4. Discussion

AK remains one of the most vision-threatening corneal infections, characterized by its diagnostic challenges, protracted course, and limited therapeutic options. The high resistance of the protozoan, particularly due to encystment, renders conventional pharmacological regimens only partially effective, frequently requiring prolonged use of biguanides and diamidines with significant ocular surface toxicity [18,19]. Against this background, preventive and adjunctive strategies targeting the earliest steps of infection are urgently needed. Our study contributes to this unmet need by providing in vitro evidence that CORNEIAL MED, a PHMB- and HA-CL–based ophthalmic solution, can reduce *Acanthamoeba castellanii* adhesion and early biofilm-like colonization of human corneal epithelium.

The reduction in adhesion observed in our experiments—approximately 33% when co-administered with trophozoites and up to 52% when applied after initial adhesion—appears modest compared with the cytotoxic effects of higher-concentration PHMB used in clinical therapy. However, these results should be interpreted in the preventive context. Even partial inhibition of epithelial adhesion may translate into clinically meaningful reductions in the risk of keratitis in susceptible populations, particularly CL wearers [20]. Indeed, epidemiological studies consistently identify lens use, combined with poor hygiene or water exposure, as the strongest risk factor for AK. A topical formulation capable of reducing early adhesion could serve as a prophylactic adjunct in high-risk scenarios such as overnight or extended wear, swimming with lenses, or post-exposure prophylaxis following accidental water contamination.

Our findings also resonate with recent in vitro investigations demonstrating amoebicidal activity of commercial eye drops not originally developed for anti-*Acanthamoeba* purposes. Lubricants such as Systane Ultra and Optiben have shown variable inhibitory effects on trophozoites [21], while anti-glaucoma drops containing timolol were reported to induce trophozoite death through mitochondrial dysfunction [22]. These data suggest that the ocular surface may be pharmacologically modulated in ways that reduce pathogen colonization. In this landscape, CORNEIAL MED offers a rationally designed combination: PHMB at a low, tolerable concentration and cross-linked hyaluronic acid to improve residence time and epithelial compatibility [23,24]. The ability of HA-CL to enhance mucoadhesion and support epithelial recovery further strengthens the case for its dual protective role.

From a translational standpoint, the product may have relevance beyond CL wearers. Patients undergoing corneal refractive surgery, keratoplasty, or surface ablation procedures may represent another population at risk of opportunistic infections during the epithelial healing phase [19]. Topical prophylaxis with a safe PHMB-based lubricant could potentially decrease the likelihood of early colonization by environmental pathogens, including *Acanthamoeba*. Moreover, in hospital settings, prophylactic use might be considered in immunocompromised patients or in those with corneal trauma requiring extended epithelial recovery [18].

Nevertheless, several limitations of the present work must be acknowledged. First, our experiments focused on trophozoites rather than cysts, which are more resistant and clinically relevant in chronic AK. Second, the reconstructed human corneal epithelium model, although standardized and widely used, cannot fully replicate the complexity of the in vivo ocular surface, including tear film dynamics, immune responses, and mechanical lens interactions. Third, our experiments employed a single PHMB concentration and a fixed application paradigm, leaving unanswered questions about dose–response relationships, frequency of administration, and long-term tolerability.

Future research should therefore extend these findings in several directions. While the observed inhibition is encouraging, these in vitro findings must be validated in ex vivo tissue and in vivo models—including cyst-stage assays—before any clinical inferences can be drawn. Pharmacokinetic studies will be essential to determine the residence time and bioavailability of PHMB at the corneal surface when delivered within an HA-CL matrix. It would also be valuable to explore combination strategies, such as pairing low-dose PHMB with antifungal or antiviral agents, to broaden antimicrobial coverage while maintaining safety. Finally, this in-vitro study evaluated a single PHMB 5 ppm, HA-CL–containing solution and did not include head-to-head testing versus other PHMB-based products. Given the known heterogeneity in anti-amoebic performance among multipurpose systems, future work should adopt a standardized, ISO-aligned protocol to compare formulations under identical conditions. Accordingly, the present results should be interpreted as product-specific and hypothesis-generating.

In summary, our results highlight the potential of CORNEIAL MED as a preventive or adjunctive tool in the management of AK. By targeting the earliest stages of pathogenesis, namely adhesion and biofilm initiation, this formulation addresses a critical gap in current therapeutic strategies. While further work is required to validate its clinical utility, these findings represent a step toward safer and more practical interventions aimed at reducing the burden of this challenging corneal infection.

## 5. Conclusions

CORNEIAL MED eye drops demonstrated the ability to reduce *Acanthamoeba castellanii* adhesion to human corneal epithelium in vitro. When administered simultaneously with amoebae, the product showed a measurable though not statistically significant reduction in adhesion. More importantly, when applied after an initial adhesion period, CORNEIAL MED produced a significant inhibitory effect, suggesting that the product can interfere with early stages of pathogen colonization and biofilm formation. Taken together, these findings indicate that even at the minimum tested dose (20 µL), CORNEIAL MED may contribute to the prevention of AK by limiting amoebic attachment to the corneal surface. These data support a preventive, in-vitro proof-of-concept for attenuating early epithelial adhesion; further ex vivo and in vivo studies are warranted to establish clinical relevance.

## Figures and Tables

**Table 1:** Percentage of reduction in *A. castellanii* adhesion with respect to the HCE + AC control.

**CORNEIAL MED Eye Drops**	**HCE + TP + AC**		**HCE + AC + TP (3 h)**	
	**Mean**	**SD**	**Mean**	**SD**
	33.0	11	51.9 *	6.5

* *p* value < 0.05.

## Data Availability

The original contributions presented in this study are included in the article. Further inquiries can be directed to the corresponding author.

## Abbreviations

The following abbreviations are used in this manuscript:

AK	*Acanthamoeba* keratitis
CL	Contact lens
PHMB	Polyhexamethylene biguanide
HCE	Human corneal epithelium
HA	Hyaluronic acid
